# Correlates of quality of life and mental health among youth experiencing homelessness in Iran

**DOI:** 10.1186/s40359-023-01145-y

**Published:** 2023-04-13

**Authors:** Neda Malekmohammadi, Soheil Mehmandoost, Mehrdad Khezri, Hossein Mirzaei, Fatemeh Tavakoli, Ghazal Mousavian, Mansureh Safizadeh, Abedin Iranpour, Hamid Sharifi

**Affiliations:** 1 HIV/STI Surveillance Research Center, WHO Collaborating Center for HIV Surveillance, Institute for Futures Studies in Health, Kerman University of Medical Sciences, Kerman, Iran; 2 Department of Epidemiology, New York University School of Global Public Health, New York, NY USA; 3 Department of Population and Family Health, Kerman University of Medical Sciences, Kerman, Iran

**Keywords:** Quality of life, Mental health, Homelessness, Youth Experiencing Homelessness, Iran

## Abstract

**Background:**

Quality of life (QOL) and mental health among marginalized populations, including people experiencing homelessness in Iran, are understudied. We assessed the QOL and mental health status as well as their associated factors among youth experiencing homelessness in Kerman, Iran.

**Method:**

We recruited 202 participants using a convenience sampling method from 11 locations, including six homeless shelters, three street outreach sites, and two drop-in service centers, from September to December 2017. Data were collected using a standardized questionnaire that included QOL, mental health, demographics, drug use, and sexual behaviors questions. Scores in each domain were indexed with a weight of 0-100. The higher score indicated a higher QOL and mental health status. Bivariable and multivariable linear regression models were performed to examine correlates of QOL and mental health.

**Result:**

The mean (SD) score of QOL and mental health were 73.1 (25.8) and 65.1 (22.3), respectively. Multivariable analysis showed that youth experiencing homelessness who were aged 25–29 years old (β = -5.4; 95% CI: -10.51; -0.30), and lived on the streets (β = -12.1; 95% CI: -18.19; -6.07) had a lower mental health score. Moreover, those who had higher education (β = 5.4; 95% CI: 0.58; 10.38), had no history of carrying weapons (β = 12.8; 95% CI: 6.86; 18.76), and had a higher QOL score (β = 0.41; 95% CI: 0.31; 0.50) had a higher mental health score.

**Conclusion:**

This study highlights that QOL and mental health among youth experiencing homelessness in Iran are concerning, particularly among those who were older, were less educated, were living on the street, and had a history of carrying a weapon. Community-based programs, including mental health care and affordable housing are needed to improve QOL and mental health among this population in Iran.

## Introduction

Homelessness is a significant public health concern facing many youths around the world [1]. The United Nations defines homelessness as absolute and relative homelessness [2], suggesting that in addition to people who do not have a permanent shelter, people who have a residence but do not meet the standard housing conditions are also considered as people experiencing homelessness [3, 4]. A growing body of evidence suggests that youth experiencing homelessness, who are between 13 and 25 years old, are at a higher risk for a wide range of social, economic, and physical and mental health problems, such as violence, substance use, and premature death [1, 5–7].

Quality of life (QOL) is an indicator with various dimensions of health, such as physical, mental, social, cognitive function and role, and general well-being [8, 9]. QOL among youth experiencing homelessness may be related to a variety of factors, including sociodemographic factors, clinical, social, and utilization of health care services [8]. Evidence suggests that the QOL among youth experiencing homelessness is lower than that of young people in the general population due to a range of adverse problems, such as substance use, violence, and other adverse mental and physical health problems (e.g., HIV and hepatitis) [10]. Moreover, studies have shown that the prevalence of mental disorders among youth experiencing homelessness is higher than that of the general population. For example, a study suggested that youth experiencing homelessness experience serious mental illnesses, such as schizophrenia, bipolar disorder, major depression, and suicide attempts [11].

In Iran, evidence suggests that the number of youth experiencing homelessness has increased in recent years. Moreover, adverse health outcomes and high-risk behaviors are also prevalent among youth experiencing homelessness in Iran. For example, studies documented that half of youth experiencing homelessness in Iran reported recent substance use [12]. High-risk sexual practices, such as unprotected sex were also reported to be common among this population in Iran [13]. However, our understanding of the QOL and mental health outcomes among youth experiencing homelessness in Iran remains limited. Therefore, we aimed to examine the QOL and mental health outcomes and their associated factors among youth experiencing homelessness in Kerman, Iran.

## Method

### Settings and study design

We recruited 202 youth experiencing homelessness from September to December 2017, in Kerman in southeastern Iran. Participants were recruited using convenience sampling through street-based outreach and referral from peers from 11 locations, including six homeless shelters, three street outreach sites, and two drop-in service centers. Eligible criteria included being between the ages of 15 and 29, being homeless or living in an unstable situation for at least one month in the previous 12 months [2, 13].

### Data collection and measures

A trained staff collected data using a standardized questionnaire through face-to-face interviews. The questionnaire was in Farsi and consisted of sections on demographic characteristics, housing status, sexual behaviors, substance use, QOL, and mental health.

We used the European Quality of Life 5 Dimensions 3 Level Version (EQ-5D-3 L) questionnaire, which is a general tool that can be used to check health and the effect of treatment on a range of diseases. This questionnaire contains five single-question dimensions that assess mobility status, self-care, usual activities, pain or discomfort, and anxiety or depression in three levels, with having no problem, having some problem, and having a lot of problem as response options [14, 15]. To convert the scores into a 0-100 index, first all questions were weighted inversely so that higher scores indicate better QOL, then the scores of the questions were added together and converted into a 0-100 index using Eq. 1. The validity and reliability of the questionnaire were assessed by Zare et al., and the Cronbach’s alpha of the questionnaire was 0.87 [16].

General Health Questionnaire – 28 (GHQ-28), which is self-report screening measure to detect possible psychological disorder, was used to assess mental health. The GHQ-28 recognizes two major issues: (1) inability to perform routine functions; and (2) the emergence of new and disturbing phenomena. The GHQ-28 is based on the original General Health Questionnaire, which had 60 items. The GHQ-28 comprises of 28 questions that are used to determine whether a person’s current mental state deviates from that state normally. The GHQ-28 includes four subscales: somatic symptoms (items 1–7), anxiety/insomnia (items 8–14), social dysfunction (items 15–21), and severe depression (items 22–28). Scores in each domain were indexed with a weight of 0-100, and the closest indicator to 100 shows better mental health [17, 18]. To convert the scores to a 0-100 index, first all the questions were weighted inversely so that higher scores indicate better mental health, then the scores of the questions were added together, and finally, using Eq. 1, the scores were converted to a 0-100 index. The validity and reliability of the questionnaire were checked in the study by Nazifi et al., and the overall Cronbach’s alpha of the questionnaire was 0.92 [19].

Covariates included sociodemographic, behavioral and structural level variables, including gender (women or men), age (15–24 or 25–29), current marital status (single, married or divorced), education level (less than high school or high school and more), nationality (Iranian or non-Iranian), average monthly income (70 USD or less, more than 70 USD, or I have no income), current living situation (living with parents, living with relatives, living with spouse, living with friends, or living on the street), lifetime history of alcohol consumption (yes or no), lifetime history of sexual contact (yes or no), lifetime drug use (yes or no), and lifetime history of carrying a weapon (yes or no).

### Statistical analysis

To calculate the QOL and mental health scores, we combined the questions related to each topic and then converted the scores to a scale of 0 to 100. The normality of the quantitative data was examined using the Kolmogorov-Smirnov and the results showed that all of them had a normal distribution. Bivariable and multivariable linear regression models to examine factors associated with QOL and mental health. Variables with a P value < 0.2 in bivariable analysis were entered into the multivariable model [20]. The final model was fitted using backward elimination method with significance was set at P value < 0.05 for retention. Statistical analysis was performed using SPSS software version 26.

## Results

### Participants characteristics

Of 202 participants, 109 (54.0%) were men, and 146 (72.3%) were 25–29 years old. The majority of participants were married (n = 128, 63.4%), had less than high school education (n = 135, 66.8%), were Iranian (n = 190, 94.1%), had monthly income less than 70 USD (n = 119, 58.9%), and lived with their spouses (n = 113, 55.9%). Overall, 67 (33.2%) ever consumed alcohol, 177 (87.6%) had a history of sexual contact, and 110 (54.0%) ever used illicit drugs (Table 1).

**Table 1:** Demographic characteristics of among youth experiencing homelessness in Kerman, Iran in 2017 (n = 202)

Variables		n	%
**Age**	15–24	56	27.7
	25–29	146	72.3
**Gender**	Men	109	54.0
	Women	93	46.0
**Marital status**	Single	60	29.7
	Married	128	63.4
	Divorced	14	6.9
**Education**	Less than high school	135	66.8
	High school or more	67	33.2
**Monthly income**	Less than 70 USD	119	58.9
	More than 70 USD	42	20.8
	I have no income	41	20.3
**Nationality**	Iranian	190	94.1
	Non-Iranian	12	5.9
**Current living situation**	Living with parents	24	11.9
	Living with relatives	10	5.0
	Living with spouse	113	55.9
	Living with friends	6	3.0
	Living on the street	41	20.3
	Living with sexual partner	8	3.9
**Lifetime history of alcohol consumption**	Yes	67	33.2
	No	135	66.8
**Lifetime history of sexual contact**	Yes	177	87.6
	No	25	12.4
**Lifetime history of drug use**	Yes	110	54.0
	No	93	46.0
**Lifetime history of carrying a weapon**	Yes	41	20.3
	No	161	79.7

### Factors associated with quality of life

The overall mean (SD) score of QOL was 73.08 (25.83) out of 100. Bivariable linear regression model showed that QOL score was significantly lower among youth experiencing homelessness who aged between 25 and 29 years (β = -8.2; 95% CI: -16.19; -0.29), were men (β = -6.3; 95% CI: -13.5; -0.83), lived on the streets compared to those who lived with their parents (β = -18.02; 95% CI: -30.74; -5.29). QOL score was significantly higher among youth experiencing homelessness who were married compared to single (β = 9.6; 95% CI: 1.82; 17.49), had high school or more education (β = 8.4; 95% CI: 0.94; 16.03), had no history of alcohol use (β = 7.8; 95% CI: 0.25; 15.43), did not have a history of carrying a weapon (β = 17.1; 95% CI: 8.40; 25.80), and had a higher mental health score (β = 0.72; 95% CI: 0.59; 0.85) (Table 2). In the final multivariable model, mental health score (β = 0.70; 95% CI: 0.57; 0.83) was the only covariate which had a significant association with QOL score among young people experiencing homelessness (Table 3).

**Table 2:** Mean score and bivariable linear regression analysis of quality of life among youth experiencing homelessness in Kerman, Iran in 2017 (n = 202)

Variables		Mean (SD)	Crude		
			B	95% CI	P value
**Quality of life score**		73.08 (25.83)	-	-	-
**Gender**	Women	76.48 (25.66)	1		
	Men	70.14 (25.73)	-6.3	13.5; 0.83	0.08
**Age**	15–24	79.02 (24.08)	1		
	25–29	70.78 (26.19)	-8.2	-16.19; -0.29	0.04
**Current marital status**	Single	67.59 (27.18)	1		
	Married	77.25 (24.12)	9.6	1.82; 17.49	0.01
	Divorced	58.04 (27.12)	-9.5	-24.35; 5.25	0.20
**Education**	Less than high school	70.25 (25.91)	1		
	High school or more	78.74 (24.90)	8.4	0.94; 16.03	0.02
**Monthly income**	Less than 70 USD	72.04 (25.40)	1		
	More than 70 USD	73.52 (25.78)	-1.4	-8.18; 9.67	0.86
	I have no income	75.61 (27.52)	-4.52	-14.76; 22.36	0.68
**Nationality**	Iranian	72.49 (25.85)	1		
	Non-Iranian	82.3 (24.69)	9.8	-5.33; 24.94	0.20
**Current living situation**	Living with parents	77.09 (24.63)	1		
	Living with relatives	77.5 (26.22)	0.41	-18.13; 18.96	0.96
	Living with spouse	77.66 (24.28)	0.57	-10.50; 11.64	0.91
	Living with friends	70.84 (25.81)	-6.2	-28.74; 16.24	0.58
	Living on the street	59.07 (26.40)	-18.02	-30.74; -5.29	0.006
	Living with sexual partner	62.50 (26.72)	-14.5	-34.70; 5.53	0.15
**Lifetime history of alcohol consumption**	Yes	67.81 (24.22)	1		
	No	75.65 (26.28)	7.8	0.25; 15.43	0.04
**Lifetime history of sexual contact**	Yes	72.95 (25.70)	1		
	No	74 (27.22)	1.06	-9.85; 11.97	0.84
**Lifetime history of drug use**	Yes	73.1 (26.61)	1		
	No	73.07 (25.68)	0.39	-0.19; 0.98	0.18
**Lifetime history of carrying a weapon**	Yes	59.38 (26.36)	1		
	No	76.48 (24.62)	17.1	8.40; 25.80	< 0.001
**Mental health score**			0.72	0.59; 0.85	< 0.001

**Table 3:** Bivariable linear regression analysis of mental health among youth experiencing homelessness in Kerman, Iran in 2017 (n = 202)

Variables		Mean (SD)	Crude		
			B	95% CI	p-value
**Mental health score**		65.15 (22.33)			
**Sex**	Women	69.60 (19.01)	1		
	Men	61.30 (24.30)	-8.3	-14.54; -2.05	0.009
**Age**	15–24	71.39 (18.42)	1		
	25–29	62.68 (23.30)	-8.7	-15.63; -1.78	0.01
**Current marital status**	Single	59.18 (24.04)	1		
	Married	68.60 (21.07)	9.4	2.43; 16.39	0.008
	Divorced	58.54 (21.06)	-0.64	-13.59; 12.30	0.92
**Level of Education**	Less than high school	62.10 (21.14)	1		
	High school or more	71.36 (23.55)	9.2	2.64; 15.87	0.006
**Average monthly income**	Less than 70 USD	62.86 (23.31)	1		
	More than 70 USD	66.03 (19.92)	3.1	-4.96; 11.32	0.44
	I have no income	70.90 (21.03)	8.6	-7.39; 24.74	0.28
**Citizenship**	Iranian	64.99 (22.59)	1		
	Non-Iranian	67.60 (18.63)	2.6	-10.55; 15.76	0.69
**Current living situation**	Living with parents	73.85 (21.68)	1		
	Living with relatives	61.89 (26.05)	-11.9	-27.87; 3.95	0.14
	Living with spouse	70.15 (20.21)	-3.6	-12.86; 5.47	0.42
	Living with friends	59.73 (22.88)	-14.1	-32.70; 4.46	0.13
	Living on the street	48.38 (20.76)	-25.4	-36.19; -14.73	< 0.001
	Living with sexual partner	52.96 (13.01)	-20.8	-37.51; -4.26	0.01
**Lifetime history of alcohol consumption**	Yes	58.98 (22.10)	1		
	No	68.27 (21.88)	9.2	2.69; 15.87	0.006
**Lifetime history of sexual contact**	Yes	64.40 (22.46)	1		
	No	70.49 (21.11)	6.09	-3.50; 15.68	0.21
**Lifetime history of drug use**	Yes	65.02 (24.98)	1		
	No	65.19 (21.61)	0.38	-0.12; 0.89	0.13
**Lifetime history of carrying a weapon**	Yes	48.25 (21.87)	1		
	No	69.27 (20.49)	21.02	13.61; 28.43	< 0.001
**Quality of life score**		----------	0.53	0.44; 0.63	< 0.001

### Factors associated with mental health

The mean (SD) score of mental health was 65.15 (22.33). Bivariable model showed that mental health score was significantly lower among youth experiencing homelessness who were men (β = -8.3; 95% CI: -14.54; -2.05), and lived on the streets compared to those who lived with their parents (β = -25.4; 95% CI: -36.19; -14.73). Mental health score was significantly higher among those who were married (β = 9.4; 95% CI = 2.43; 16.39), had high school or more education (β = 9.2; 95% CI: 2.64; 15.87), had no history of alcohol consumption (β = 9.2; 95% CI: 2.69; 15.87), had no history of carrying a weapon (β = 21.02; 95% CI: 13.61; 28.43), and had a higher QOL score (β = 0.53; 95% CI: 0.44; 0.63) (Table 4). In multivariable linear regression model, youth experiencing homelessness who were aged 25–29 years old (β = -5.4; 95% CI: -10.51; -0.30), and lived on the streets (β = -12.1; 95% CI: -18.19; -6.07) had a lower mental health score. Moreover, youth experiencing homelessness who had higher education (β = 5.4; 95% CI: 0.58; 10.38), had no history of carrying weapons (β = 12.8; 95% CI: 6.86; 18.76), and had a higher QOL score (β = 0.41; 95% CI: 0.31; 0.50) had a better mental health status (Table 3).

**Table 4:** Multivariable linear regression analysis of quality of life and mental health among youth experiencing homelessness in Kerman, Iran in 2017 (n = 202)

Quality of life				
Variables		**B**	**95% CI**	**p-value**
**Mental health score**		0.7	0.57; 0.83	< 0.001
**Mental health**				
**Age**	15–24	1	--	--
	25–29	-5.4	-10.51; -0.30	0.03
**Education**	Less than high school	1	--	---
	High school or more	5.4	0.58; 10.38	0.02
**Current living situation**	Living with parents	1	--	--
	Living with relatives	5.8	-5.63; 17.21	0.31
	Living with spouse	-2.1	-14.22; 9.97	0.72
	Living with friends	6.9	-6.40; 20.21	0.30
	Living on the street	-12.1	-18.19; -6.07	< 0.001
	Living with sexual partner	6.8	-5.67; 19.44	0.28
**Lifetime history of carrying a weapon**	Yes	1	--	--
	No	12.8	6.86; 18.76	< 0.001
**Quality of life score**		0.41	0.31; 0.50	< 0.001

## Discussion

These findings demonstrated that the level of QOL and mental health status are concerning among youth experiencing homelessness in Kerman, Iran. Furthermore, Multivariable analysis for mental health showed that youth experiencing homelessness who were older, were less educated, were living on the street, had a history of carrying a weapon, and had a lower QOL had a lower mental health level.

Our estimate for QOL status among youth experiencing homelessness in Iran was comparable with QOL of people experiencing homelessness living in other countries, such as Canada [21], and Germany [22]. Moreover, our estimate for QOL status among youth experiencing homelessness in Iran was lower compared to the general population in Iran [23]. Existing literature suggests an association between homelessness and life satisfaction which could affect the QOL [24]. We found that youth experiencing homelessness with lower QOL had worse mental health status. Previous studies conducted in Quebec, Canada and Oklahoma, United States also have shown that the mental health disorders among people experiencing homelessness could result in lower QOL [21, 25]. These findings suggest that improving the QOL and mental health among youth experiencing homelessness in Iran are needed. This underscores the need for effective mental health services, providing affordable housing, and employment to improve the QOL among youth experiencing homelessness.

Our estimate for mental health score among youth experiencing homelessness in Iran were also comparable to the estimates for mental health in studies conducted in other countries, such as Canada [1] and United States [26] Additionally, the mental health score among youth experiencing homelessness in this sample was lower than that of the general population in Iran [23, 27]. The results of the regression model suggested a significant association between mental health score and living on the streets. The findings of previous cross-sectional study conducted in Spain has showed a similar association and the odds of sleeping on the street was five times higher for the homeless women who had mental health problems [11]. People experiencing homelessness who live on the streets are usually struggling for basic needs, such as shelters, nutrition, and fundamental health-related services which increase the level of stress and anxiety for them and could result in mental health problems [13, 28]. Studies found that providing housing for people experiencing homelessness with mental health disorders could decline the risk of hospitalization because of mental health disorders over time [29, 30]. Consequently, providing shelters and housing for these people could be considered as an approach for improving mental health among youth experiencing homelessness in Iran. Providing mental health services through the community and street-based out-reach services are also warranted.

Our study has several limitations that should be acknowledged. First, data were collected among youth experiencing homelessness in three street outreach sites, six homeless shelters, and also two service centers; therefore, the results may not be generalized to all youth experiencing homelessness in Kamran, Iran. Second, social desirability, under-reporting, and recall biases may be present given that behavioral data were collected through face-to-face interviews. Lastly, the cross-sectional nature of study limits the ability to conclude causal and temporal relationships.

## Conclusions

We found that youth experiencing homelessness in Iran dealt with poor QOL and mental health outcomes. There is a need for mental health services to help youth experiencing homelessness cope with various mental health problems and increase their QOL. Moreover, multilevel prevention interventions at both the individual and community levels, including programs aimed at creating resilience in young people to better deal with stressors on the streets and programs to address risk factors for mental health disorders, should be provided for youth experiencing homelessness in Iran.

## Data Availability

Data are owned by the Ministry of Health (MOH) of Iran and are available for researchers who meet the criteria for access to confidential data. The authors of this research were the implementers of the survey and had access to the data with permission obtained from the MOH. The data are available from the corresponding author on reasonable request.
